# From Waste to Wealth: Unlocking the Potential of Cellulase Characteristics for Food Processing Waste Management

**DOI:** 10.3390/foods14213639

**Published:** 2025-10-24

**Authors:** Muhammad Hammad Hussain, Kamran Ashraf, Redhwan Ebrahim Abdullah Alqudaimi, Maria Martuscelli, Shao-Yuan Leu, Salim-ur Rehman, Muhammad Shahbaz Aslam, Zhanao Li, Adnan Khaliq, Yingping Zhuang, Meijin Guo, Ali Mohsin

**Affiliations:** 1 State Key Laboratory of Bioreactor Engineering East China University of Science and Technology, 130 Meilong Road, Shanghai 200237, China; sheikh.hammadhussain786@gmail.com (M.H.H.); redwanalqudaimi@gmail.com (R.E.A.A.); ypzhuang@ecust.edu.cn (Y.Z.); guo_mj@ecust.edu.cn (M.G.); 2 Department of Bioscience and Food, Agricultural and Environmental Technology, University of the Studies of Teramo, Via Balzarini 1, 64100 Teramo, Italy; 3 Department of Civil and Environmental Engineering, The Hong Kong Polytechnic University, Hong Kong, China; syleu@polyu.edu.hk; 4 Research Centre for Resources Engineering Towards Carbon Neutrality (RCRE), The Hong Kong Polytechnic University, Hong Kong, China; 5 Research Institute for Future Food (RiFood), The Hong Kong Polytechnic University, Hong Kong, China; 6 Department of Food Science and Technology, Riphah International University Faisalabad Campus, Faisalabad 38000, Pakistan; drsalim_rehman@yahoo.com; 7 School of Biochemistry and Biotechnology, University of the Punjab, Quaid-e-Azam Campus, Lahore 54590, Pakistan; 8 Institute of Food Science and Technology, Khwaja Fareed University of Engineering and Information Technology, Rahim Yar Khan 64200, Pakistan

**Keywords:** food processing wastes, cellulase, agroindustrial-based biorefineries, genetic engineering, fermentation technology, bioremediation

## Abstract

A surge in environmental pollution compels society to utilize food processing wastes to produce valuable compounds. Enzymatic technology, specifically cellulase-mediated hydrolysis, provides an eco-friendly and effective approach for treating food processing leftovers. The main objective of this review is to explore the significant contributions of cellulase, both in industrial settings and from an environmental perspective. Therefore, this review covers all the aspects of cellulase structural identification, classification, and evolution to its profound applications. The review initially explores cellulases’ structural and functional characteristics based on the catalytic and cellulose-binding domains and discusses cellulases’ evolutionary origin. A thorough understanding of cellulase properties is essential for overcoming the challenges associated with its commercial production for various applications. In this regard, the optimization for cellulase production through several approaches, including rational design, direct evolution, genetic engineering, and fermentation technology, is also reviewed. In addition, it also underscores the significance of agro-industrial biorefineries, which provide scalable and sustainable solutions to meet future demands for food, chemicals, materials, and fuels. Finally, the last sections of the review solely highlight the potential applications of microbial cellulases in bioremediation. In summary, this review outlines the role of cellulase in efficient valorization aimed at producing multiple bioproducts and the enhancement of environmental remediation efforts.

## 1. Background

Much of the food processing waste is rich in cellulose, hemicellulose, and lignin, simply known as cellulosic biomass. This waste has risen considerably due to multiple factors, including urbanization, high population growth, and increased demand for agricultural and industrial products. The most notable food processing wastes are pectin-rich fruit waste, rice straw, sugarcane bagasse, and cotton stalk, which are known to have numerous applications in food and medical applications. *Candida bombicola* and *Candida tropicalis* are known to consume food processing wastes due to their high biomass and functionality. They use waste frying oil and corn cob, respectively, to produce sophorolipid biosurfactants and xylitol [1,2]. The cell wall of food processing residue consists of polysaccharides that can be a carbon source for various microorganisms, and their improper management leads to environmental pollution [3]. This review discusses the urgent need for several bioproducts in our community by determining the significance of enzymatic technology in valorizing agroindustrial wastes. This innovative method not only increases resource utilization but also contributes to sustainable bioprocessing. As a result, there has been an unprecedented global focus on every aspect of cellulase enzymes, including their structure and functional characteristics related to the hydrolysis of cellulose-rich food processing waste.

Cellulase enzymes facilitate the breakdown of cellulose during hydrolysis. Due to extensive hydrogen bonding, cellulose establishes a durable crystalline structure capable of enduring for millions of years at neutral pH without enzymes. Cellulolytic microorganisms participate in the degradation of cellulose-rich waste materials, which contribute approximately 8% of global greenhouse gas emissions through three primary mechanisms [4]. The first mechanism is utilized by aerobic organisms, including *Trichoderma reesei*, which secretes free cellulases [5]. The second mechanism involves anaerobic bacteria, such as *Clostridium thermocellum*, that employ large multienzyme complexes known as cellulosomes. Finally, some organisms, such as *Cytophaga hutchinsonii* (aerobic soil bacterium) and *Fibrobacter succinogens* (anaerobic rumen bacteria), remain tightly attached to the cellulose substrate, and their degradation strategies’ intricacies are poorly understood.

The breakdown of cellulosic biomass is driven by three major enzymes: endoglucanase, exoglucanase (cellobiohydrolase), and β-glucosidase. Even though these enzymes serve unique functions, they work synergistically to transform cellulose into glucose effectively [6]. Endoglucanase enzymes cleave the amorphous region of cellulose randomly to generate cello-oligosaccharides and create new chain ends. Following this, exoglucanase enzymes act on the ends of these chains to produce cello-oligosaccharides, cellobiose, and glucose. This process is initiated by endoglucanases, which target the amorphous areas and generate access sites for exoglucanases to reach the crystalline region. Lastly, the β-glucosidases hydrolyze cellobiose into glucose, thus preventing the product inhibition of cellobiohydrolases [7]. All cellulolytic enzymes feature a “substrate binding site” in a distinct carbohydrate-binding domain and an “active site” in the catalytic domain. Most cellulases possess carbohydrate-binding domains (CBDs) and catalytic domains (CDs). This review offers a comprehensive analysis of these crucial domains and highlights the evolutionary significance of cellulase. Understanding cellulase’s structure and functional properties allows genetic modifications and mutations in the cellulase enzyme.

These advancements have boosted the applicability of cellulase enzymes across multiple industries over the past few decades, including biorefinery, food, detergent, pharmaceutical, paper, textiles, and more [8]. According to the United Nations Environment Programme (UNEP), the waste stream consists of food and organic waste, which account for 47% of the world’s annual waste generation, as well as paper and plastic waste roughly make up 17% and 12% of all waste, respectively [9]. In another study conducted in Bangladesh, it was found that residential waste has a higher proportion of food waste (59.5%) than that of the industrial sector, which produces a lower quantity of food waste, approximately 26–30% [10]. The different proportion of waste generation is thought to be a result of variation in the consumption patterns and commercial activities across different sectors worldwide. This waste accumulation has a negative impact on the environment, causing water and soil contamination, greenhouse gas emissions, and leachate formation. These waste-related problems are the main driving factor for the elevated demand for cellulase enzymes. As of 2022, the global cellulase market was valued at approximately USD 1.621 billion and is projected to reach USD 2.45 billion by 2026 [11]. Regardless of its massive growth, there are several knowledge gaps identified in the biotechnological applications of cellulases. The most important ones are the challenge of low yield and high substrate cost in the enzyme production at the commercial level. Additionally, there is a lack of detailed studies on enzyme–substrate interactions, which limits the development of thermostable cellulases. Other challenges include lignin-induced difficulties in enzymatic hydrolysis and the limited experience with cellulase-based systems in functional biorefineries. However, the genetic engineering of microbial strains with efficient enzyme production and optimized fermentation appears to be an effective way to address these gaps that lead cellulase biotechnology to sustainable industrial applications.

This review highlights the need to focus on biotechnological production and strategies to improve enzyme properties and yields. Additionally, these optimized enzymes support bioremediation and effectively mitigate pollution. Unlike previous works, this review presents a detailed analysis of cellulase structure, classification, and evolutionary aspects, as well as its contribution to waste management. It uniquely relates the enzyme evolution with its multifaceted contribution in food and non-food sectors, which provides an interesting context for both practical bioprocess and fundamental science.

## 2. Basic Structural and Functional Features of Cellulase

Carbohydrate-active enzymes (CAZymes) are involved in both the synthesis and hydrolysis of complex polysaccharides. Cellulases, the most common CAZymes, consist of a CBD and a CD essential for their activity. CBDs contain binding sites to form an interaction with cellulose, and the CD has a catalytic site where the chemical reaction proceeds. The CD executes important hydrolysis for degrading food processing waste, whereas the CBDs help anchor the enzyme onto this cellulose-rich substrate, leading to higher enzyme concentration on the insoluble cellulose surface. CBDs connect to CDs through a flexible linker rich in proline and threonine [12]. These linkers are believed to promote close interactions between the biocatalyst and its challenging substrate, thereby enhancing catalysis. Hence, the CBDs are essential for the initiation and processivity of enzymes such as exoglucanases [13]. The removal of CBD from the enzymatic subunit significantly diminishes its activity. Conclusively, the CBD performs a vital function of locating and securing the enzyme onto cellulose for optimal functioning of CD, and this effective CD-CBD duo allows continuous chain cleavage necessary for treating the solid food processing waste.

Fungal cellulase has a relatively more uncomplicated structure than bacterial cellulase, as it contains cellulosome cohesion-bearing enzymes for scaffolding and dockerin. Scaffoldin (Sca), also known as a scaffolding protein, is the functional unit of the cellulosome, and it consists of a cellulose binding domain, cohesins, an S-layer (SLH) module, X modules, and a dockerin [14]. The cohesion modules comprise approximately 150 amino acid residues and are present as tandem repeats in the scaffolding protein. The cellulosomal complex results from the interaction of the dockerin modules with the cohesin modules. The dockerin domain comprises about 70 amino acids and has two 22-residue duplicated segments. The dockerin and cohesion specificity for their modular counterpart and modular arrangement on the Sca subunit dictate overall cellulosome architecture. Cellulase also has other domains like the Nod B-like domain, S-layer homologous domain, and fibronectin-type III domain [15].

## 3. Classification and Annotation of Cellulase

**Figure 1:**
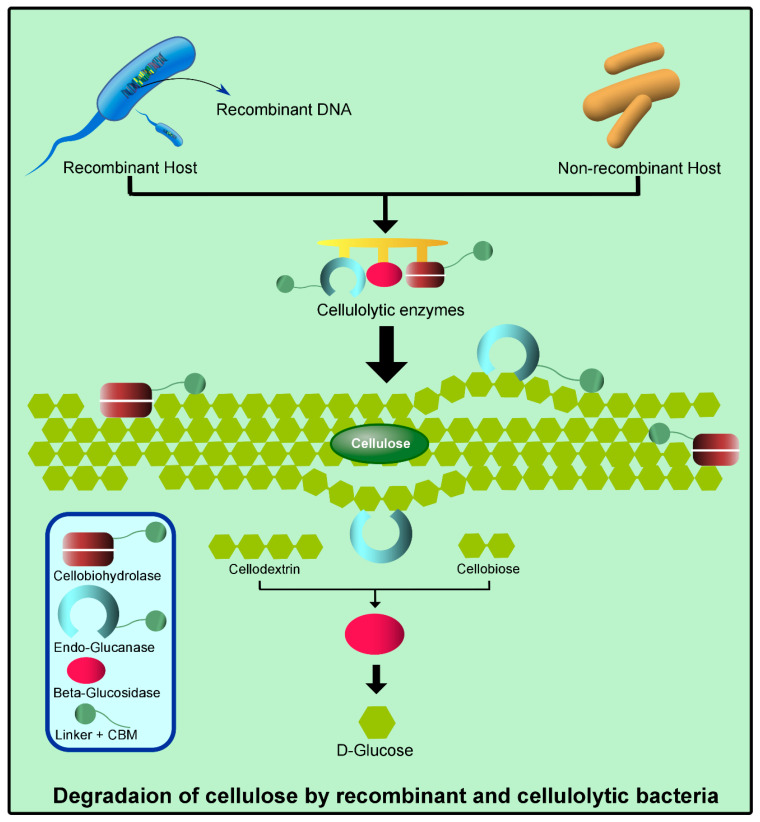
Degradation of the complex cellulosic structure by the cellulolytic and non-cellulolytic organisms.

Recent advances in annotation systems for cellulose-active enzymes have improved the process of assigning functions to newly identified cellulases. As these conserved enzymes within CAZy families operate harmoniously in a tailored cascade, which empowers them to overcome the structural complexity of food processing waste. According to the CAZy database, the members of all three cellulase classes (endoglucanases, exoglucanases, and β-glucosidases) belong to different glycoside hydrolase (GH) families. Their classification is prepared based on the depolymerization stage of the targeting substrate [16]. According to the cellulase structure and sequence of amino acids, the catalytic modules are categorized into distinct hydrolase families. CD sub-classification is attributed to various complex, fold-oriented structures (lectin-like β-sandwich, 8-barrel, and 6-barrel type) and amino acid sequences [17]. However, structure-based classification categorizes catalytic domains into 15 of more than 80 known GH families during the 1990s [18]. These families are 5/A, 6/B, 7/C, 8/D, 9/E, 10/F, 11/G, 12/H, 26/I, 44/J, 45/K, 48/L, 51, 60, and 61. Furthermore, *β*-amylases and glucoamylases are classified in families 14 and 15, respectively. On the contrary, the CBMs consist of 30–180 amino acid residues, which exist as a single, double, or triple domain in one protein. CBMs can be classified into types: Type A, B, and C. Type A CBM has been assigned a function to recognize the crystalline polysaccharides and bind to them. Type B CBMs bind the internal glycan chain. These binding sites appear as extended clefts comprising binding subsites to accommodate long sugar chains. Type C CBMs bind to short oligosaccharides externally via pocket-shaped binding sites [19]. In the past five years, fifteen new families of CBMs have been discovered. More so, other kinds of auxiliary proteins, like lytic polysaccharide monooxygenase, assist in the oxidative cleavage of the crystalline cellulose bond, which enormously improves the cellulase efficiency against recalcitrant waste streams. These enzymes can be produced by naturally cellulolytic isolates (belonging to *Thermobifida*, *Trichoderma*, and *Pseudomonas*) and engineered microbial systems (*Escherichia* and *Bacillus*) as illustrated by Figure 1, maximizing the opportunity to turn the problem of managing cellulose-rich food processing waste into useful bioproducts.

## 4. Evolution of Cellulase

**Figure 2:**
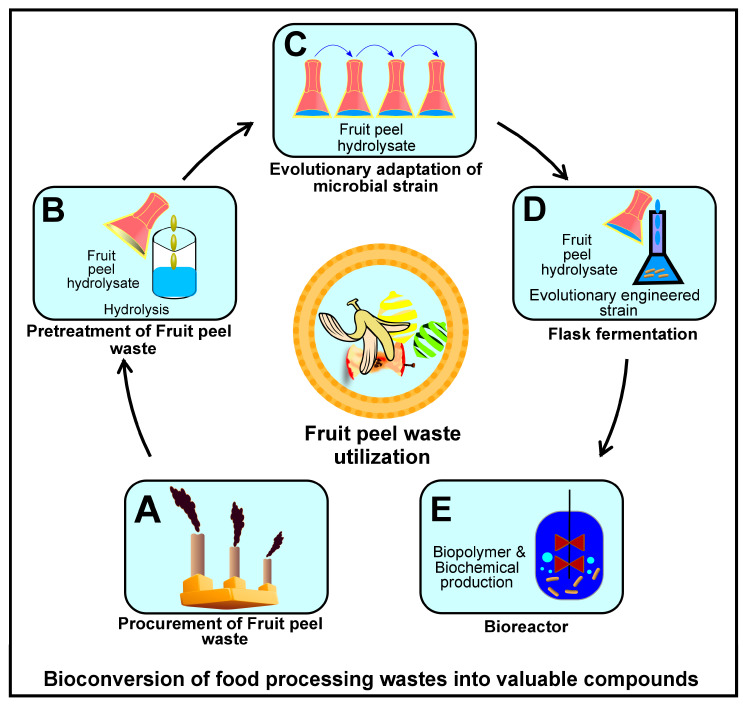
Bioconversion of food processing wastes into several bioproducts.

Environment-friendly products, like biopolysaccharides and biochemicals, can be synthesized from food processing wastes through targeted microbial conversion, as shown in Figure 2 [3]. However, only select lineages of bacteria and fungi are reported to perform the bioconversion task efficiently. The evolutionary processes that bring about these modifications have been investigated by transcriptomic and biochemical analyses of biomass-degrading microbes at the single-cell level [20]. For example, a comparison of the phylogenetic diversity of over 1100 strains of *Streptomyces* was carried out, and it further revealed two distinct clades (I and III) within 29 highly cellulolytic strains. Interestingly, up to 93% of strains in these two clades were isolated from diversified eukaryotic hosts, while the remaining strains were from diverse ecological niches and geographical regions (soil and marine) [21]. These evolutionary processes produce a robust microbial system to deconstruct cellulose in agroindustrial and waste environments, which is fundamental for effective carbon cycling and bioremediation. Many methods have been identified to transmit microbial symbionts from parent to offspring: coprophagy, cytoplasmic inheritance, direct contact during and after birth, etc. The transmission of *GH5* and *GH45* genes from the common ancestor of insects to the coleopterans, either by horizontal transfer or vertical transfer is still unclear [22]. The findings from other studies indicate that substitution in critical residues of target specimens may lead to different substrate specificity, such as horizontally transferred *GHF9* genes to sea squirts, which acquired a new activity to hydrolyze chitin instead of cellulose, as well as its activity is also detected in both angiosperms and a single amoebozoan [23,24]. In addition, strong evidence for the horizontal transfer of cellulase genes between bacteria and animals has been reported. Table 1 examines the transfer of genes between different organisms of the same or other species.

## 5. Strategies for Cellulase Improvement

High production costs for cellulase enzymes impede their utilization, especially in various everyday sectors grappling with high waste challenges in food and non-food sectors. Therefore, various strategies, such as mutagenesis, genetic engineering, and fermentation technology, have been exploited to increase the economic feasibility of cellulolytic enzyme production at a commercial level. Numerous reports show advancements in cellulase activity, as seen in Table 2, underscoring its significance.

### 5.1. Rational Design Approach

Traditionally, the rational design is used to design a protein. However, this technique is pursued after developing the recombinant DNA (rDNA) technique and site-directed mutagenesis (SDM). The rational design consists of three steps: enzyme selection, identification of the target amino acid site that needs modification, and mutant characterization [55]. In this context, exposure to physical or chemical mutagenesis is beneficial in generating improved strains, including *T. viride* N879, *Bacillus* sp. C1 mutant C1M26, *Aspergillus* sp. XTG-4, and *Cellulomonas* sp. TSU-03 mutant M23 [56]. Cellulase-producing mutants have low cellulase yield, and the mutagenesis procedure is challenging and time-consuming. So, rational strategies are required to modify cellulase production. This technique optimizes the activity, enhances the stability, improves enzyme specificity, and aids in product development. More so, site-directed mutagenesis is a potent tool for altering secondary structure and amino acid sequence. It can also facilitate fusion protein development; even the whole domain can be exchanged. *Thermotoga maritima Cel5A* (endoglucanase) is engineered for better thermostability by CBM modification and site-directed mutagenesis [57]. Through this modification, *Cel5A* becomes more robust and efficient for field-scale applications. Furthermore, the enzyme can be altered for the production of 3-methyl-1-butanol (longer-chain alcohol) for its better conversion rate into biofuel [58]. Thus, the conversion of cellulose-rich refuse into biofuel directly replaces fossil fuels and fulfills society’s needs.

### 5.2. Directed Evolution Method

In directed evolution, the cellulolytic enzymes undergo an iterative selection and mutation cycle to improve their activity and function. This approach can increase the stability, efficiency, and specificity of cellulase enzymes, which promote their industrial applications. The direct evolution method is a multi-stage laboratory process by which microorganisms with desired characteristics are created. This method combines the random mutagenesis of genes with DNA shuffling, error-prone PCR, and a staggered extension process with high-throughput screening and selections. The success in directed evolution is mainly dependent on the size of the library, as the larger library allows the various mutants to be evaluated, and it increases the chance of picking the best mutant with the desired characteristics. Recently, Noguchi et al. [59] found the impact of random mutagenesis (mutagenesis by ultraviolet ray (UV) and N-methyl-N′-nitro-N-nitrosoguanidine (NTG)) on *T. reesei* to yield cellulase that showed better β-glucosidase activity (54 UmL^−1^) and filter paper assay activity (15.6 UmL^−1^) on lactose substrate with enhanced production (119 g/L) [59]. This development of a mutant strain by Noguchi et al. offers an efficient and affordable solution for the processing of agro-industrial waste. In another study, the cellulase production was increased approximately to 182 FPU/g with a decrease in medium viscosity by exposing *T. reesei* (RUT-C30) to two mutagenic agents, diethyl sulfate and UV [60].

Several reports show that mutagenized strains are attributed to more desired characteristics when compared to parental strains. Chand et al. [61] found that the strain *A. niger* CMV5-A10 became more robust after being treated with 1-methyl 3-nitro-1-nitrosoguanidine, and a two-fold increase in cellulase yield was noticed [61]. Similar findings have been reported by Singh et al. [62], who demonstrated that a mutant of *Aspergillus* sp. Su14 was developed by sequentially treating fungal spores with γ-irradiation of NG, Co60, and UV [62]. Compared to the parental strain, the mutant *Aspergillus* sp. SU14-M15 strain resulted in an 8.5-fold increase in the production of cellulase enzymes [62]. Therefore, random-based strain modification is frequently used to improve cellulase production and enzyme activity by many folds. When these optimized strains are cultured in scalable bioreactors, it leads to enhanced cellulose degradation. It further increases bioremediation by converting food processing waste to fermentable sugar, which enables resource recovery and mitigates greenhouse gas emissions. Computer-guided rational methods, such as molecular mechanics calculations, docking studies, simulations, and quantum mechanics calculations, use various types of computational technologies. These methods take less time to analyze enzyme properties and screen numerous compounds simultaneously. This pre-screening process can assure the selection of superior enzyme variants for pilot-scale remediation trials. On the contrary, semi-rational engineering is created by coupling directed evolution with computational methods. Hence, both these methods (rational components and computer prediction) are used in unison to overcome the major limitations of the two approaches. It analyzes the data from mutation studies to design scaffolds and enzyme active sites [63]. More so, this method evaluates the change after the directed evolution, and this data, together with the structure-function relationship information, is highly effective in designing cellulolytic enzymes that steer us towards a sustainable ecosystem.

### 5.3. Genetically Engineering the Strain

In recent times, genetic engineering has influenced cellulase production quality so that it can actively degrade the cellulosic food waste. It can be achieved through numerous approaches, such as cellulase gene overexpression, optimization of promoter sequence, and modification of negative regulators. In one of the studies, the cellobiohydrolase I (*Cbh I*), an inducible promoter gene, is employed for heterologous protein production of *T. reesei*. For instance, Haakana et al. [64] investigated the impact of two endoglucanases and one CBH (isolated from *Melanocarpus albomyces*) and their expression in *T. reesei* under the CBHI promoter on the production of target products [64]. The boosted enzyme level under the strong promoter improves the degradation of cellulose and cost-effectiveness in the valorization of wastes. In another study, da Silva Delabona et al. [65] reported the 26-fold constitutive overexpression of the *xyr1* gene in *T. harzianum* using a pki1 promoter, which further resulted in the enzymatic complex formation [65]. The resulting complex increased the hydrolysis rate of bagasse substrate by 25%. It can be assumed that the quick breakdown of bagasse into soluble sugar improves the feedstock quality for anaerobic microorganisms. Another successful genetic engineering approach is to modify cellulase CBM to design a chimeric enzyme with high hydrolytic activity. In chimeric enzyme synthesis, one enzyme’s catalytic domain is fused with another domain (CBM) of another species. Genetic engineering aims to generate thermotolerant and thermostable chimeric proteins with enhanced substrate specificity. This modification makes it ideal for extreme environments such as sludge heaps and composts [66]. Similar findings have also been reported by Park and coworkers et al. [67], who isolated a thermostable exoglucanase gene from *Streptomyces* sp. M23 to clone and express in *S. lividans* TK-24 to make it stable up to 100 °C [67]. It can be further suitable for high-temperature applications, such as anaerobic digestion and composting. Similarly, Li et al. [68] reported the isolation of the thermostable endoglucanase gene from *B. subtilis* to clone and express in *E. coli*, which led to threefold higher enzyme activity [68].

Genetic engineering can improve the inherent abilities of cellulase enzymes, such as activity, specificity, stability, and productivity. Seiboth et al. [69] found that the removal of lae1 methyltransferase protein eliminates the cellulase activity in *T. reesei*. However, the *laeI* gene’s overexpression enhanced transcript levels and the activity of the cellulase enzyme [69]. The importance of an engineered consortium in improved cellulase production has been investigated in parallel with the monoculture system. In one study, it has been identified that the *ClrB* (activator) ortholog from *P. oxalicum* and *CreA* (repressor) are key transcription factors that induce the expression of the cellulase enzyme gene. The trigenic recombinant strain RE 10 is generated by overexpressing *ClrB* and deleting *CreA* and *bgl2*. The resulting strain RE 10 exhibited a high cellulase enzyme secretion, together with a 20-fold increase in filter paper activity [70]. This versatility is critical for addressing mixed waste streams in the food processing industry. Under the systems biology approach, the overexpression of 28 regulatory genes from *T. reesei* was thoroughly studied to evaluate the optimal condition requirements for increased cellulolytic enzyme production [71]. Both cloning and expression of bacterial and fungal cellulase genes to improve cellulase enzyme production in different hosts are shown in Table 2.

### 5.4. Improving Cellulase Production Through Microbial Fermentation

During fermentation, the cellulase and xylanase enzymes convert food-processing wastes into useful products, like biofuel, biogas, etc., to enhance resource efficiency. The feedstock materials for fermentation should be productive and inexpensive, such as rice straw, husk, wheat bran, sugar cane bagasse, etc. Several external factors, including temperature, pH, substrate concentration, etc., affect cellulase production, enzymatic activity, structural stability, and glucose yield [72]. The cellulolytic enzyme production is a cost-representative step. Two major technologies, including solid submerged fermentation (SmF) and solid state fermentation (SSF), are currently employed to produce cellulase, pectinase, and proteases by microbes. Especially, the SSF technology is employed to boost cellulase production, which further aids in bioremediation and waste management. According to the economic analysis, the unit cost to produce cellulase was approximately $40.36 and $15.67 per Kg for SmF and SSF. The price deflation and inflation calculation by a factor is 0.9 and 1.1. Both of these methods are capable of meeting local and industrial needs.

#### 5.4.1. Solid State Fermentation (SSF)

SSF has been widely applied to economically produce industrially important cellulase enzymes through cellulosic biomass. Moreover, it operates with simple equipment that requires low energy and water to yield a high level of enzymes, thereby reducing the processing cost and wastewater generation. Recycling solid substrates (nutrient-rich waste) is an effective and common way of growing microorganisms in the SSF process. More so, no foam formation during the SSF process makes it an ideal technique for enhanced enzyme production. Multiple cellulolytic organisms such as *B. licheniformis*, *Bacillus* sp., *B. cereus*, and *Clostridium thermocellum* are cultivated by using the SSF approach, which further transforms agro-wastes into environmental and economic assets. For example, Cavka et al. [73] found that the engineered *A. niger* expressing *Cel7B* cellulase was grown on waste fiber sludge from pulp mills to produce ethanol [73]. Moreover, Moran-Aguilar found that the SSF technique led to the highest cellulase activity, approximately 6.23 U/g, by *Aspergillus* species on bagasse and brewery grain [74]. Similarly, the *Acremonium cellulolyticus* C1 was directly cultivated on the paper sludge (PS) to generate up to 40 g/L of cellulosic ethanol within 80 h at flask scale [75]. The spent mushroom substance was also used as a substrate to achieve cellulase activity of 18.82 U/mL by *A. niger* in SSF [76]. Compared to SmF, cellulase produced by *Trichoderma harzianum* on cardboard waste exhibited 21% more cellulase activity in SSF [77]. At the industrial scale, the different SSF bioreactors like fluidized bed, rotary drum, tray, stirred-aerated, and packed bed bioreactors play a significant role in waste management by allowing controlled degradation of sludge and food processing wastes using cellulase. The production of cellulolytic enzymes through solid-state fermentation on food processing wastes is shown in Table 2.

#### 5.4.2. Submerged Fermentation (SmF)

Interest in submerged fermentation is sharply increasing, and the optimization of various factors such as aeration rate, agitation speed, temperature, foam formation, and pH is necessary for sustainable production and improved microbial growth. Often, the supernatant obtained from SmF comprises crude cellulase cocktails to treat the food processing wastes. At the industrial scale, submerged fermentation has served as a well-established technology due to the easy recovery of enzymes and precise control of the system. For instance, the thriving of *Bacillus subtilis* on grape pomace and *Trichoderma reesei* on pea hull produces robust cellulase-rich culture broth with a specific activity of 0.178 U/mL and 13.8 U/mL, respectively [78,79]. SmF-derived enzymatic hydrolysis increases many-fold the degradation at soil remediation sites, anaerobic digester, and compost piles, thus the pollutant load is reduced. For example, SmF-driven *A. niger* culture is applied to treat food processing-derived wastewater and sludge under optimized conditions, and the resulting enzyme broth led to ~95% of turbidity reduction and ~97% of COD removal [80]. At commercial scale, a bubble column reactor with a volume of 3L has been employed to produce cellulase by *Trichoderma harzianum* from domestic wastewater, and it is reported to have a high cellulolytic activity of 645 U/L.h [81]. After investigating lab and pilot scale studies, it has been reported that the fermentative production of cellulase is complicated and depends on several parameters, including the design of reactors, fermentation conditions, types of biomass, and microorganisms. In some cases, SmF utilizes molasses or exopolysaccharides to produce enzymes, which create an environmental barrier or adsorb pollutants [82]. Various reports regarding cellulase production in submerged fermentation are depicted in Table 2.

In emerging studies, unproductive cellulase adsorption was found to be a challenging situation that limits the efficiency of cellulase-based enzymatic saccharification. As a result, the cellulolytic activity is reduced. The addition of non-catalytic protein (soybean and bovine serum albumin) and non-ionic surfactant has thus been investigated for resolving this lignin-mediated enzyme reduction by increasing cellulolytic activity with low enzyme loading [83]. The use of surfactant reduces enzyme deactivation at the air–liquid interface, which facilitates hydrolysis in complex waste slurry. In addition, the independent physicochemical factors are evaluated by a response surface methodology in two notable strains, *Aneurinibacillus aneurinilyticus* and *Schizophyllum commune* COC, which led to optimized cellulase production [84]. In a study, cellulase production is enhanced in *Bacillus licheniformis* KY962963 by optimizing the environmental and nutritional parameters through the Plackett–Burman” design and one factor at a time approach [85]. Subsequently, the statistical modeling determines factors that positively affect cellulase production during fermentation. The elevated enzyme yield accelerates biomass degradation that leads to decreased food processing waste buildup, which ultimately supports sustainable bioprocessing and bioremediation.

## 6. Industrial Applications of Microbial Cellulase

**Figure 3:**
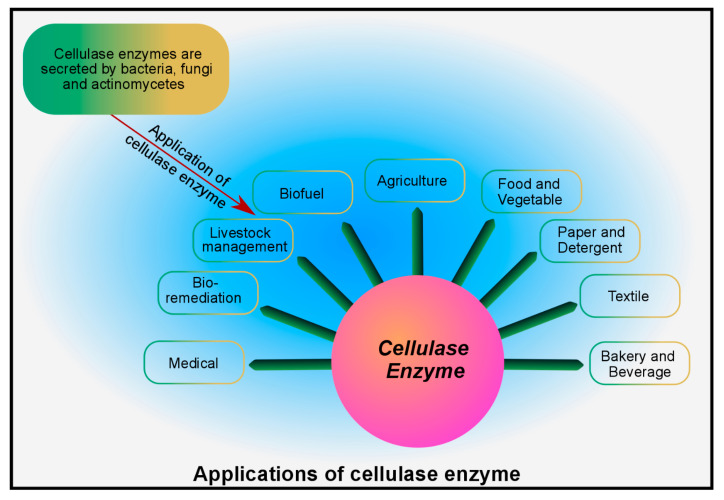
Role of cellulase enzymes in different industry sectors [87].

Cellulose is the most abundant and cost-effective renewable energy resource with an estimated annual biomass production of around 1.5 × 10^12^ tons globally [86]. It is the main polysaccharide of food processing waste, and its conversion to glucose requires cellulase enzymes. Cellulases have been commercially available for over 30 years and are now coupled with fermentation engineering to convert cellulosic biomass into novel bio-products at a commercial scale. These cellulases are being used in various industries around the globe, such as food (as functional additives), biofuels (for the production of bioethanol and biogas), agriculture, textiles, laundry, chiral separation, ligand binding, and waste management, as shown in Figure 3 and Table S1 [87].

Interestingly, recent studies have shown some advantages of cellulase application in addition to the well-known disadvantages, such as lower enzyme stability, high production cost, substantive substrate pretreatment, and inhibition of product, which prompts the decreased catalytic efficiency [88,89]. These benefits include eco-friendliness, extensive resource valorization, reduced energy demand, and mild operational requirements during the transformation of waste into valuable products [90,91]. Cellulase-related biotechnological processes are now more economically viable mainly due to advances in genetic engineering and fermentation technology, as well as in process optimization, enzyme immobilization, and enzyme engineering.

## 7. Role of Microbial Cellulase in Waste Management

The exposure to environmental pollution is a major threat to public health worldwide. In 2012, the generation rate of municipal solid waste (MSW) by 3 billion people was around 1.2 kg/capita/day. According to a World Bank report, this rate is expected to rise to around 1.42 kg/capita/day of MSW by 2025 for 4.3 billion urban residents [92]. More importantly, the mismanagement of these wastes has been proven to cause several environmental problems, such as the release of offensive odor, the occurrence of infectious diseases, and contributions to climate change. On a dry weight basis, most of the typical wastes generated by domestic and industrial sectors are rich in various biopolymers, including cellulose (28.8 to 54.3%), hemicelluloses (6.6 to 11.9%), and lignin (12.1 to 28%) [93]. Therefore, the bioconversion of MSW by aerobic and anaerobic treatments in the presence of cellulase has led to biofuel, biogas, and compost production, and it turns this waste management issue into a revenue opportunity at national and sub-national levels.

### 7.1. Bioconversion Through Ex Situ and In Situ Bioremediation Techniques

**Figure 4:**
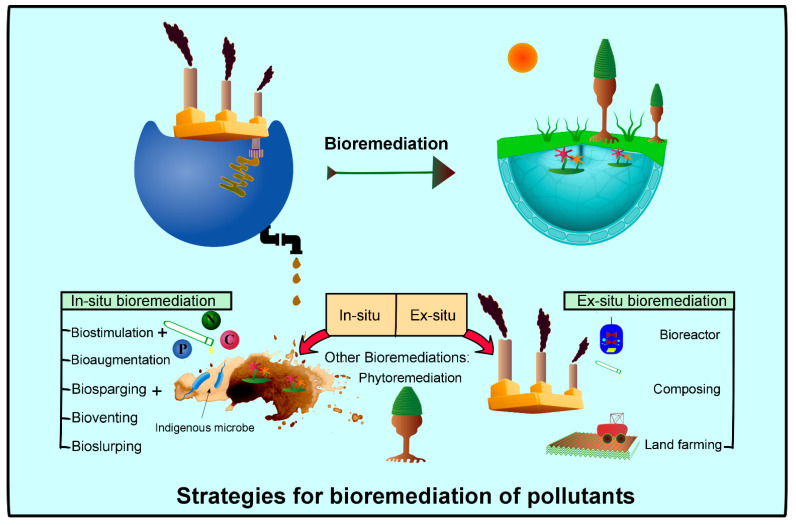
Treatment of various wastes via In situ and Ex situ bioremediation [94].

There are two major bioremediation processes: ex situ bioremediation and in situ bioremediation, as presented in Figure 4 [94]. In the review, we discussed some ex situ bioremediation techniques (composting and anaerobic digestion) and in situ bioremediation techniques (phytoremediation and floating treatment wetland), which participate in cleaning up contaminants in soil and groundwater.

#### 7.1.1. Composting

Composting is an environmentally friendly approach as it effectively recycles the organic waste secreted from municipal and industrial plants. It can improve soil quality and health by remediating polluted soil, controlling plant diseases and erosion, and promoting the growth of soil microbes. Soil erosion and anthropogenic activities lead to the rapid loss of soil organic matter in farmlands, whereas in some areas the conversion of primary forests to agricultural croplands reduces soil organic carbon in the topsoil by about 58% [95]. Composting techniques have been investigated to resolve the problem of organic waste management. Organic waste like municipal refuse consists of higher values of macro- and microelements such as Ca, Mg, Zn, NaOH, Al_2_O_5_Si, and KOH, and lower levels of nitrogen and phosphorus content. By using microbial inoculants, the biological decomposition is increased, which further facilitates the conversion of this organic waste into nutrient-enriched compost [96]. The experiments were mostly conducted with *Aspergillus* sp., *Trichoderma* sp., and *Penicillium* sp. to produce cellulase in the organic waste degradation process [97]. Currently, Gram-negative and Gram-positive bacteria, such as *Streptomyces* sp., *Bacillus* sp., *Clostridium* sp., and *Pseudomonas* sp., are cultivated and grown for the production of cellulolytic enzymes. These enzymes bind to their cell wall and can degrade native lignocellulose preparation effectively [97]. As an example, Awasthi et al. [97] found that co-composting MSW combined with sludge and mixed microbial culture (e.g., *Candida rugopelliculosa*, white-rot fungi, *Lactobacillus buchneri*, and *Bacillus casei*) led to the highest degree of mineralization rate and a lower value of nitrogen loss [97].

*Chaetomium thermophilum* fungus is isolated from municipal waste compost to secrete laccases required for producing polyaromatic humic substances associated with peroxidase and phenoloxidase. Two bacterial isolates (CRCB9 and CRCB10) of *C. panacarvi* producing mannanase, β-glucanase, cellulase, and xylanase for efficient degradation of cellulose were isolated from coffee residue compost (CRC) [98]. Rastogi et al. [99] stated that *Cohnella* sp. has been reported to degrade sawdust, carboxymethyl cellulose, and cellulose, and some species of the genus *Cohnella*, including *C. damensis* sp., *C. thailandensis* sp., and *C. panacarvi* sp., displayed good xylanolytic activity [99]. Similarly, Sizova et al. [100] found that the compost consisted of horse manure, food waste, and wood chips can be a good source to isolate anaerobic bacteria (*Clostridium* sp.) capable of degrading cellulose and xylan [100]. In another study, Kato et al. [101] isolated the anaerobic cellulolytic bacterium, *Clostridium straminisolvens*, from its microbial community [101]. Hence, the coexistence of cellulolytic and hemicellulolytic bacteria (anaerobes) can be linked to the process of composting.

Many studies demonstrated that compost-associated microbes have been used to alter soil microbial activity and composition and promote soil fertility and health [102]. The favorable processing parameters, including a C/N ratio (25–35), temperature (50–55 °C), moisture content (50–60%), and pH (5.5–8.0), support the growth and activity of soil microbes. Application of omics technology has been proposed to determine the link between the composition and diversity of soil microbial communities and their ecological functions. In addition, a plant mixture can remediate soil contaminated with heavy metals through adsorption, precipitation, and redox reactions [103]. More so, it was found that metallo-regulatory proteins control the expression of different genes that allow various microorganisms (i.e., *Escherichia coli* and *Cornyebacterium diphtheriae*) to cope with heavy metal toxicity [104]. Table S2 demonstrates the role of soil microbes in promoting development and enhancing soil quality and bioremediation during composting.

#### 7.1.2. Anaerobic Digestion

Microbial consortia containing cellulolytic bacterial and fungal communities, such as *Trichoderma*, *Bacillus*, and *Ochrobactrum*, have been found to possess high substrate degradation activities [105]. This method has been shown to generate an increased biogas production of up to 38% [105]. However, additional waste valorization techniques, such as chemical, biological (aerobic and anaerobic digestion of effluent), and bio-electrochemical systems (BES), should also be considered to address environmental pollution facing our societies [106]. Among these techniques, anaerobic digestion (AD) is predicted to be one of the most promising methods due to its ability to convert numerous waste effluents and food processing residuals (FPRs) into valuable compounds such as methane. Anaerobic digestion of these cellulosic wastes shows a low methane production rate, entailing long solid retention time, and requires a large reactor size for enhanced methane production [107]. In aerobic digestion, the hydrolysis of cellulosic and hemicellulosic components of food processing residues is the rate-limiting step, that are catalyzed by microbial-derived cellulase and xylanase.

The pretreatment of cellulase and amylase plays a significant role in utilizing cellulose and starch for methane production. Anaerobic digestion is characterized by high methane production and a short lag phase. For instance, Ziemiński and Kowalska Wentel stated that the anaerobic co-digestion of sugar beet pulp and vinasse via enzymatic pre-treatment increases methane yield by 57.7% [108]. Similar findings have been reported by Karray et al. [108], who demonstrated that the pretreatment of *Ulva rigida* with pure β-glucosidase at 50 °C for 2 h resulted in a substantial increase in biogas yield up to 626.5 mL/g COD [109]. Zhao et al. [110] found that the corn straw pretreatment with enzyme T isolated from the *T. harzianum* culture can promote the biogas yield to 273.75 mL/g·TS [110].

Bioaugmentation of mixed cultures has also been found to increase the production of biogas by 15% during the anaerobic digestion of sweet corn processing residues [111]. Kupski et al. [112] determined the hydrolytic activity of cellulolytic complex in achieving protein and starch digestion, which was beneficial to boost hydrolysis during the anaerobic digestion of corn straw [112]. Weide et al. [113] investigated the impact of an enzyme mixture on agricultural waste to produce methane. However, after 60 days of BMP tests, the increase in methane production (−2.7% to 9.4%) was not detectable [113]. Advanced techniques such as 16S rRNA gene-sequencing provide tools to determine the phylogenetic relationship and function of the active microbial community that reflects the anaerobic digestion process and observe changes in the microbial community in response to different processing conditions. Optimum processing conditions, such as temperature, pH, and waste-to-inoculum ratio, are critical for maintaining the optimum methanogenic bacteria activity in anaerobic digestion. For instance, Regueiro et al. [114] found that the biological pretreatment (for 12 h) and microbial hydrolysis of cassava residues in a batch bioreactor (at 55 °C) led to the optimum production of methane up to 259.46 mL/g-VS [114]. Table S2 represents various hydrolytic microbes and their communities used in anaerobic digestion to generate biogas and digestate through biomass waste.

#### 7.1.3. Phytoremediation and Floating Treatment Wetland

Phytoremediation is an appealing technique that involves the use of plants (i.e., *Brachiaria mutica*, *Zea mays*, *Nicotiana glaucum*, and *Helianthus annuus*), microorganisms, and enzymes. The most representative enzyme classes, such as transferases, oxidoreductases, hydrolases, and dehalogenases, gained growing popularity in remediating polluted environments. The plant and plant-microbe interactions are the main producers of these enzymes. Compared to different contaminant remediation methods, phytoremediation is more economical by 5–13 times. Depending on pollutant types, various mechanisms participate in phytoremediation, such as degradation, stabilization, extraction, volatilization, and filtration. However, the efficacy of phytoremediation is influenced not only by the soil or land type and level of biotic and abiotic conditions, but also by the pretreatment of heavily polluted sites before planting. Despite this, the operating expenses of the phytoremediation method are comparable to or even lower than many other physical and chemical methods [115]. Floating treatment wetland (FTW) technology has become a popular way to treat wastewater more effectively. FTW is a soilless planting technology that removes pollutants through adsorption, sedimentation, and biodegradation.

Heavy metal contamination in water and soil can induce stress and cause damage to plants. Various factors influence the translocation of accumulated metals in plants, like microbial activity, water pH, type of plant species, and their associated enzymes [116]. Identifying the litter decomposition characteristics and microbial communities is necessary to protect the area affected by heavy metal pollution. *Basidiomycota* can produce cellulase and lignin-modifying enzymes (LMEs) and are widely applied to control litter decomposition. For instance, Jia et al. [117] identified enzymatic activities and bacterial community characteristics of *Imperata cylindrica* litter as the phytoremediation progressed. It has been noticed that the concentration of catalase and cellulase enzymes is lower in *Imperata cylindrica* litter initially; however, as the phytoremediation progresses, the bacterial communities encoded with peroxidase and hemicellulase are gradually dominated [117]. More so, it is found that the activity of laccase and cellulase is negatively affected by lead (Pb) stress in *Phyllostachys pubescens* litter [117]. Most endophytic bacteria can develop resistance against heavy metals through various mechanisms, including transforming contaminants into less toxic substances, active efflux of metal ions, and sequestration of metal ions. For example, the *Pseudomonas aeruginosa* inoculation leads to a significant increase in cadmium (Cd) tolerance in plants, which can result from the enhanced translocation of Cd in inoculated plants [118].

Transgenic plants secrete various kinds of enzymes to degrade the toxic pollutants in the rhizosphere. In one case, the transgenic *Arabidopsis* plant expresses aromatic-cleaving extradiol dioxygenase (DbfB) to degrade 2,3-dihydroxybiphenyl (2,3-DHB) [119]. Similar findings have been reported by Uchida et al. [120], who demonstrated that the transgenic tobacco plant expresses haloalkane dehalogenase (DhaA) to catalyze the detoxification of 1-chlorobuatne (1-CB) in the rhizosphere [120]. In addition, *Azospirillum* (rhizospheric bacteria) resides in the rhizosphere, where it stimulates auxin, a plant hormone, affecting the pH and enhancing the nitrogen fixation process [121]. Some contaminants have such a high level of toxicity that they lower microbial diversity and biomass, which causes a reduction in phytodegradation and further decreases the phytoremediation efficiency in the phytoremediating plants. Finally, releasing root exudates, adding various amendments, increasing contact between contaminants and roots, and using native, mixed, and transgenic species can resolve the problem of plants being site-specific and reduce environmental pollution. Table S2 describes the role of indigenous or non-indigenous bacteria in the plant-assisted bioremediation of polluted soil or water.

### 7.2. Integrated Application of In Situ and Ex Situ Cellulase Bioremediation

The integration of in situ and ex situ bioremediation through cellulase effectively degrades the food processing waste in a versatile and efficient way. In the in situ method, the contaminated biomass, including sludge, compost piles, or agricultural residues, is directly subjected to free cellulase that enables the real-time hydrolysis, reducing volume on-site and escalating the microbial decomposition. Once the cellulose-rich biomass is partially degraded, it is transferred ex situ into the compositing systems and bioreactor carrying immobilized cellulase, anchored on the supports such as metal–organic framework (ZIF-8), mesoporous silica, bio-inspired silica, or magnetic halloysite nanotubes, which promotes the high-yield saccharification under an optimized environment. This technique enables recycling of enzyme activity several times, maintaining 70–90% activity across multiple cycles [122,123,124]. For example, the cellulase immobilization on magnetic halloysite nanotubes resulted in the ~93% saccharification and a retained activity of 68% over seven cycles [122]. Similar studies have been reported by Lombardi et al. [124], who entrapped the enzyme in bio-inspired silica that maintains approximately 90% activity after five uses, along with increased pH and thermal stability [124]. Additionally, the combination of approaches, including immobilization on ZIF-8, has boosted in situ biogass hydrolysis by 93% to that of the free enzyme system and also improved tolerance to ionic liquids [125]. Other examples include the entrapping of cellulase derived from *T. reesei* on sol–gel, which led to increased resistance to glucose inhibition and retained 92% activity post-immobilization over repeated batch cycles [126]. The coupling of in situ free cellulase-mediated hydrolysis with ex situ immobilized enzyme bioreactors maximizes cellulose conversion efficiency, reduces enzyme cost, and tightens process control across residual waste.

### 7.3. Role of Cellulase Enzymes in Mitigating Global Warming and Environmental Impacts

**Figure 5:**
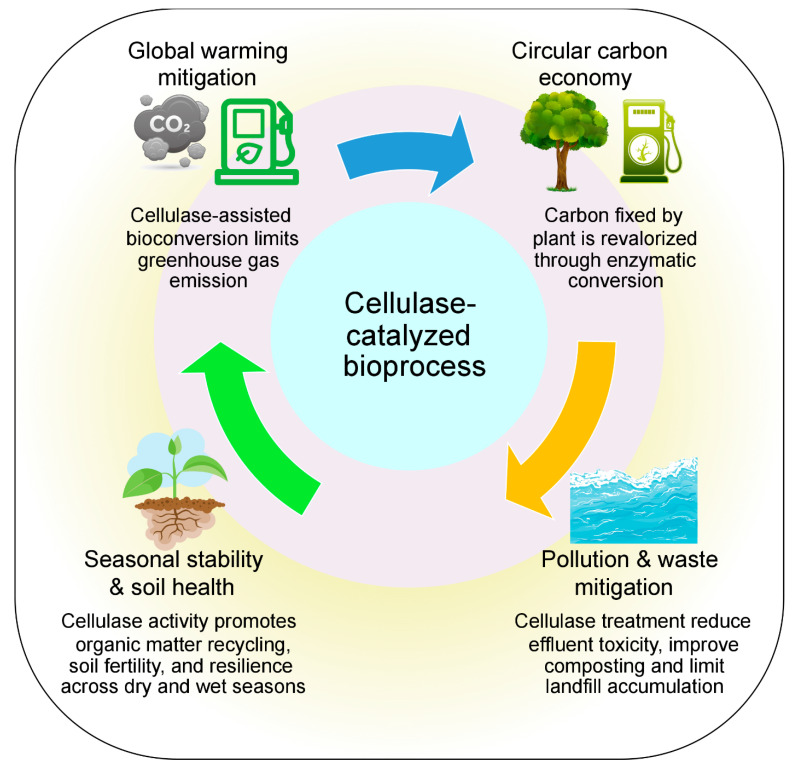
Impact of cellulase enzyme on environmental sustainability.

In general, cellulase enzymes act as biocatalysts and have been found to address several environmental issues, including global warming, ecosystem imbalance, and waste accumulation, as illustrated in Figure 5. Cellulases can turn food processing waste’s cellulose into fermentable sugar, which is then utilized to create biofuels and other renewable bioproducts. Hence, cellulase can be a competitive alternative to fossil reserves, resulting in minimized greenhouse gas (GHG) emissions. Bioethanol production in a cellulase-based biorefinery is associated with 80% lower carbon dioxide emissions than a petroleum-derived biorefinery, according to recent life cycle assessments (LCAs). Carbon neutrality is facilitated by this accomplishment through optimized cellulase-based bioethanol production [127,128]. Furthermore, compared to carbon dioxide, methane produced by the uncontrolled anaerobic breakdown of organic waste has a 28–34 times greater potential to cause global warming. Therefore, the interest in optimized cellulase-mediated biomass conversion has been sharply increasing due to the necessity of limiting methane emissions from this process [129].

Beyond the reduction of carbon emissions, cellulase enzymes preserve the environmental balance throughout the seasons. The hydrolytic ability of cellulase promotes bioremediation and composting, which further results in reduced open-field burning, increases the breakdown of lignocellulosic wastes, controls air pollution, and decreases biomass accumulation during dry seasons. The biomass waste, which is associated with off-flavor, is predominantly reduced by cellulase activity, and it also minimizes eutrophication risks in aquatic ecosystems during wet seasons by preventing anaerobic putrefaction and stabilizing organic matter [130,131].

Furthermore, it has been demonstrated that the use of cellulase in the treatment of industrial wastewater reduces total suspended solids (TSS) and chemical oxygen demand (COD), which provides a sustainable substitute for harsh chemical oxidants. Romna et al. [132] stated that the coupling of cellulase with pronase E resulted in the reduction in solids and TSS by 80% and removed particulate COD by 93% when added to sewage sludge [132]. Similar findings have also been reported by Parmar et al. [133], who demonstrated that a mixture of cellulase, protease, and lipase decreased TSS by 30–50% in sludge, and the combination of cellulase and protease is associated with superior solid reduction when compared to the outcome of individual enzymes [133]. Lastly, it is found that the cellulase enzyme largely contributes to sustainable bioprocessing and to the development of climate-resilient strategies because of its biogenic recycling mechanism. This mechanism is responsible for the maintenance of the global carbon sequestration balance.

## 8. Technoeconomic Analysis in Cellulase Biorefinery

Recently, biomass-based biorefineries have been identified as producing various biofuels and biochemicals with cellulase enzymes, which act as an integral component. The global biofuel market is estimated to expand to USD 950 million by 2024 [134]. The conversion of cellulose is at the forefront of biotechnology; however, the complexity in lignin structure hinders its potential utilization and extraction of valuable aromatic compounds [135]. Meanwhile, the higher cost of producing enzymes, which make up 20–40% of the raw materials used in lignocellulosic biorefineries, is another challenge; therefore, incorporating cellulase production directly into the biorefinery can lower costs. Moreover, the coupling of low-cost substrates with strategies like raising volumetric productivity and improving enzyme loading could further decrease the cellulase manufacturing costs. Typically, a study on techno-economic evaluation demonstrated many positive features of cellulase production, including its unit cost in solid state fermentation was roughly US $23.10/kg (batch) and US $22.12/kg (semi-batch), which is less in comparison to typical market values of US $45–60/kg [136]. Therefore, targeting lignin residues not only enables us to extract valuable chemicals but also diverts the incineration or landfill of this persistent biomass that creates environmental challenges. The rational integration of lignin valorization into cellulolytic biorefineries converts the stubborn waste into resource streams that further advance the bioremediation and circular economy [137].

The pretreatment method employed to counter lignin recalcitrance is biologically compared to chemical pretreatment. The cellulase enzymes could be adsorbed onto lignin through hydrophobic and hydrogen bonding interactions. Leu and his coworkers demonstrate the potential, pretreatment, and technology related to lignocellulosic wastes, and comprehensive reviews on lignocellulosic biorefinery are available online [137,138,139]. Shankar et al. [140] used laccase-producing *Ganoderma lucidum*, with a high level of laccase activity (~5087 U/g) to hydrolyze lignin in various food processing wastes, such as rice bran, sorghum bagasse, cotton stalk, and wheat straw [140]. Moreover, *G. lucidum* laccase has successfully executed wastewater bioremediation, including sludge treatment and dye decolorization that achieves over 95% efficiency in removing pollutants [141]. More so, Zanuso et al. [142] stated that the immobilization of cellulase enzyme onto magnetic nanoparticles significantly enhanced the hydrolysis of corn cob biomass [142]. This immobilized enzyme retains 48% of its catalytic activity, and the escalation in saccharification yield by 64% was observed after 13 cycles [142]. This biocatalytic hydrolysis ensures the greener and safer remediation of lignocellulosic waste by avoiding harsh pretreatment.

**Figure 6:**
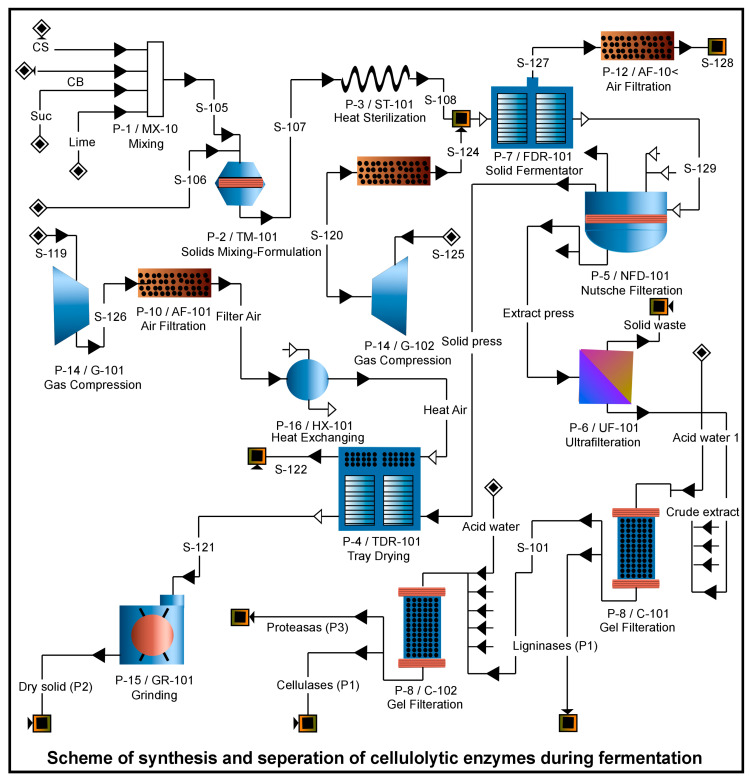
Production and isolation of cellulolytic enzymes during fermentation.

The integrated cellulosic biorefinery improves operational sustainability by limiting energy-intensive methods and harsh chemicals, which results in environmental and economic gains. Enzyme production that is fully integrated or on-site can also lower expenses related to formulation, transportation, and unnecessary capital duplication. In one compelling example, the cellulosic biorefinery is adapted for the on-site co-production of the cellulolytic enzyme from agricultural waste biomass (AWB), particularly coffee husk (Figure 6). This method avoids chemicals and delivers enzyme-driven degradation that converts waste into enzymes in situ in a single circular loop. Taiwo et al. [136] assessed the economic evaluation of cellulolytic enzyme production during batch and semi-batch processes through SuperPro Designer Software [136]. Similar findings have also been reported by Ferreira et al. [143], who conducted a techno-economic analysis of integrating β-glucosidase in engineered *Escherichia coli* to promote industrial production of low-cost enzymes, which further increases its applicability in biorefinery and real-time waste remediation [143]. In another study, Brondi et al. [144] conducted a retro-techno-economic analysis of soya bean protein used as a functional additive during saccharification in an integrated biorefinery. By decreasing the necessary enzyme loading to 5.6 FPU/g, the enzymatic hydrolysis by the waste-derived protein additives becomes more economically viable [144]. Cost-saving methods like chromatography, precipitation, and filtration during cellulase purification can further reduce costs. Compared to the batch production process, the cellulolytic enzymes produced by the continuous process are cost-efficient. For instance, Barta et al. [145] evaluated the capital cost of ethanol production (0.42–0.53 SEK/L) at the industrial level after analyzing the process design of softwood-based ethanol plants, even though 60–78% of the capital cost of cellulase production is due to the enzyme [145]. In one of the studies, the coupling of SSF with sulfite-pretreated pine slurry results in an optimized ethanol titer of 68.5 g/L. From a waste-management perspective, pine residues can be processed on-site to eliminate the requirement for enzyme transport and energy-intensive pretreatment [146]. Continuous research, process optimization, development in new operations, and technological innovations are the keys to converting food processing waste into clean energy and providing sustainable bioremediation solutions. Overall, the economic models imply that incorporating cellulase production into biorefineries can make the process viable and aid in lowering the minimum selling price of biofuels or bioproducts under the right circumstances.

## 9. Conclusions

Effective waste management is essential for sustainable agro-industrial processing, agricultural yield, and the environment’s overall health, including the well-being of the climate and people. This review examines and summarizes economically viable methods for valorizing food processing waste through enzyme-based techniques that are recognized as some of the most environmentally sustainable options available. Enzymes, especially cellulases, hold significant potential for various industries, including biofuels, detergents, paper, food, pharmaceuticals, and agriculture, and they are also used in treating various diseases. Moreover, these enzymes play a crucial role in enhancing waste management practices. This review additionally offers structural and functional insights into the various families of cellulase enzymes. Recent genetic engineering and fermentation technology advancements can improve cellulase activity for bioremediation and reduce associated costs. However, further progress in biotechnology and microbiology is still needed to harness cellulases’ potential fully.

## 10. Future Aspects

Microbial cellulases offer diverse applications, but their high production costs hinder widespread use. Significant challenges are enhancing pretreatment techniques for cellulosic biomass, devising economical production methods, and improving enzyme characteristics like stability and tolerance. Research efforts focus on reducing costs by utilizing waste materials in synthesizing enzymes and developing innovative immobilization matrices to increase catalytic efficiency. Advancements in all of these areas will promote the sustainable use of resources through energy-efficient, cheap, and environmentally friendly bioprocesses, which can transform food processing wastes into valuable bioproducts and unlock new possibilities in industrial biotechnology.

## Figures and Tables

**Table 1:** Evolution of cellulolytic gene via horizontal gene transfer and vertical transmission.

Organism	Origin	Acquired Enzyme	Significance	Reference
*Macrotermes* spp.	*Termitomyces* spp.	Exoglucanases	Both nodules and comb materials are eaten by termites. The acquired digestive enzymes play a role in the biology of fungus-feeding invertebrates.	[25]
Siricid wood wasps	Wood-rot fungi	Cellulase, Xylanase	Wood wasp larvae devoid of Cx-cellulases and the xylanases. The larvae acquire these enzymes while ingesting tissue of *Amylostereum chailletii* (fungal symbiont).	[26]
Resident gut bacterium	Marine bacterium	Agarase, porphyranase	Seaweeds with associated bacteria may have been the route through which these CAZymes are acquired in human gut bacteria (*Bacteroides thetaiotaomicron*).	[27]
Fungi and insect	*Firmicutes*, *Actinobacteria*	GH48-type enzymes	The enzymatic activity of GH48 proteins coded by horizontally transferred genes had been verified via experiments.	[28]
*Pristionchus pacificus*	Eukaryotic host	Cellulase	*Pristionchus pacificus* cellulases are embedded in a cluster of cellulases from amoeba and algae and have been reported to bring diverse resources into the ecosystem.	[29]
*Bursaphelenchus* spp.	Fungal origin	GHF45 cellulase	Nematode stylet secretes Bx-ENG-1, 2, and 3 into plant tissues. HGT made a significant contribution to the evolution of plant parasitism in nematodes.	[30]
*Aphelenchoides besseyi*	Fungal origin	GH45 cellulases	Fungi-consuming nematodes achieved the endo-1,3-β-glucanase genes from bacteria and obtained cellulase genes from fungi via HGT.	[31]
Urochordate *Ciona intestinalis*	Bacteria	Cellulose synthase gene	Ci-CesA is a fusion that consists of a cellulase domain and cellulose synthase domain, and both have no animal homologs. There is proof of likely lateral transfer of the desired gene into the urochordate lineage.	[32]
Lower termite & Cockroach Cryptocercus	Flagellate derived from parabasalid and oxymonadid lineages	GHs in flagellate	Before the evolution of eusociality, the vertical transmission of symbionts and metabolic interdependence between the host and flagellates existed. Digest cellulose via symbiotic relationship.	[33]
Sea squirts, termites, abalone	Primitive metazoan ancestor	GHF9 gene	All contain GHF9 genes with introns in identical positions, indicating that they inherited it vertically from ancient metazoan ancestors.	[34]

**Table 2:** Multiple techniques to improve the production of cellulase.

Microbial Strain	Improvement Strategies	Improved Characteristics	Reference
*Gloeophyllum trabeum*	Mutagenesis	Site-directed mutagenesis on loop 6 to improve the activity of cellulase (GtCel5)	[34]
*Coniophora puteana*	Mutagenesis	Site-directed mutagenesis was applied on β-glucosidase to enhance the enzyme activity of mutants CpBgl-A240S and CpBgl-Q20C by 58.5% and 65.7%	[35]
*Aspergillus oryzae A4*	Mutagenesis	Mutagenized via Recombinant DNA technology in which four cellulase genes, such as *cel A*, *cel B*, *cel C*, and *cel D*, are inserted, it further leads to increased secretion of cellulase and enhanced lipid production.	[36]
*Acidothermus* *cellulolyticus*	Mutagenesis	Parental strain C-1 was treated with two mutagens (UV-irradiation and NTG), and the FPase activity (17.8 U/mL) of the mutant strain CF-2612 was also increased. At the same time, its cellulase productivity by using batch culture reached 240.3 FPU/l/h.	[37]
*Trichoderma reesei RUT C30*	Mutagenesis	The six-step mutation caused mutant strain CL 847 formation and generated a two-fold increase in cellulase production compared to Rut C30.	[38]
*Thermobifida fusca*	Mutagenesis	Mutation of the conserved residue F476 to Y476 from *Cel9A* results in a 40% improvement in catalytic activity.	[39]
*Acidothermus* *cellulolyticus*	Mutagenesis	Substitution of Tyr245 to Gly (Y245G) in endocellulase Cel5A enzyme alleviates the product inhibition and results in a 40% increase in the release of soluble sugar.	[40]
β-glucosidase mutants *BGL-1*, *BGL-14*	Rational design	Surface charge is altered under applied zeta-potential gradient to improve catalytic efficiency and rate of hydrolysis by 42% and 14% in the β-glucosidase (BGL)-14 mutant.	[41]
*Penicillium verruculosum*	Rational design	The proline substitution enhances the thermal stability of cellobiohydrolase (Cel7A), and the 3.5-fold increase in the half-life of the resulting protein (G415P) was observed at 60 °C.	[42]
*Trichoderma harzianum*	Directed evolution	*Trichoderma harzianum* EU2–77 mutation using UV, NTG, and ethyl methyl sulfonate improved the activity of FPase (14.79 IU/mL)	[43]
*Trichoderma reesei* QM9414	Directed evolution	A T-DNA-tagged mutant library created by the AMT method is used to increase cellulase production in three mutants, TE-6, TA-32, and TB-87, showing 31%, 38%, and 51% increased cellulase activity compared to the parental strain.	[44]
*Trichoderma reesei*	Genetic engineering	pAMH110 vector carrying cellobiohydrolase I gene’s promoter and terminator sequences are used to enhance endoglucanase productivity by a factor of 2–4	[45]
*Brevibacillus brevis*	Genetic engineering	*Pyrococcus horikoshii’s* cellulase was cloned and expressed in *B. brevis*, resulting in a 20-fold increase in cellulase production.	[46]
*Pichia pastoris*	Genetic engineering	*T. aurantiacus’s* β-glucosidase cloned and expressed in *Pichia pastoris* to enhance its cellobiose utilization	[47]
*Trichoderma reesei*	Genetic engineering	The Pyruvate decarboxylase (*pdc*) and enolase (*eno*) promoters of *T. reesei* were used to express xylanase II and its productivity was 1.52 g/L with the *eno* promoter and 1.61 g/L with the *pdc* promoter.	[48]
*Serratia rubidaea*	Fermentation	During submerged fermentation, the microbial hydrolysis of alkaline pretreated pulpy biomass leads to significant FPase (0.5 U/mL) and xylanase (11.98 U/mL) activities (pH 8, 55 °C).	[49]
*Penicillium oxalicum* GZ-2	Fermentation	Wheat straw as an inducer enhanced β-xylosidase (89 mU/mL) and xylanase (115.2 U/mL) activities during submerged fermentation.	[50]
*Trichoderma reesei*	Fermentation	During solid-state fermentation, *Trichoderma reesei* has been reported with enhanced CMCase (8.66 U/g) and FPase (5.68 U/g) activity by using copra and wheat bran waste in a 30 L rotary fermentation.	[51]
Consortia	Fermentation	SSF of microbial consortia (*Sphingobacterium composti*, *Barnettozyma californica*, *Pseudoxanthonomas taiwanensis*, and *Cyberlindnera jardinii*) was performed in a 50 L bioreactor for improved cellulase production on coffee husk.	[52]
*Aspergillus fumigatus*	Fermentation	In SSF, the *Aspergillus fumigatus* and *Eleusine coracana* husk were used under optimized parameters of substrate concentration (1–2%), temperature (60 °C), and pH (between 2 and 4) to achieve optimal CMCase (95.2 U/mL) and β-glucosidase (0.174 U/mL) activity.	[53]
*Penicillium oxalicum*	Fermentation	In submerged fermentation, *Penicillium oxalicum* generated a 1.7-fold increase in cellulase production, with an optimal cellulase activity of 1.2 FPU/mL after optimizing processing conditions.	[54]

## Data Availability

No new data were created or analyzed in this study. Data sharing is not applicable to this article.

## Supplementary Materials

The following supporting information can be downloaded at https://www.mdpi.com/article/10.3390/foods14213639/s1: Table S1. Application of cellulolytic enzymes; Table S2. Application of microbial enzymes in waste management.

## Abbreviations

The following abbreviations are used in this manuscript:

AD	Anaerobic digestion
AWB	Agricultural waste biomass
BES	Bio-electrochemical systems
CAZymes	Carbohydrate-active enzymes
CBDs	Carbohydrate-binding domains
Cd	Cadmium
CDs	Catalytic domains
CRC	Coffee residue compost
FPRs	Food processing residuals
GH	Glycoside hydrolase
LMEs	Lignin-modifying enzymes
MSW	Municipal solid waste
Sca	Scaffoldin
SDM	Site-directed mutagenesis
SmF	Solid submerged fermentation
SSF	Solid state fermentation
UV	Ultraviolet ray
