# Supplementary material for: From Waste to Wealth: Unlocking the Potential of Cellulase Characteristics for Food Processing Waste Management
Source: Foods. 2025 Oct 24;14(21):3639. doi: 10.3390/foods14213639 (PMC12609622; doi:10.3390/foods14213639)
Supplement: Supplementary file 1 [file foods-14-03639-s001.zip › foods-3912919-supplementary.pdf]

Title

From waste to wealth: Unlocking the potential of cellulase characteristics for food processing waste management

Authors

Muhammad Hammad Hussain [1], Kamran Ashraf [1], Redhwan Ebrahim Abdullah Alqudaimi [1], Maria Martuscelli [2]***, Shao-Yuan Leu [3,4,5]**, Salim-ur-Rehman [6], Muhammad Shahbaz Aslam [7], Li Zhanao [1], Adnan Khaliq [8], Yingping Zhuang [1], Meijin Guo [1], Ali Mohsin [1] *

Table S1- Application of cellulolytic enzymes

Sector	Enzyme	Applications	Reference
Medical	Cellulase	It could be an effective alternative to antibiotics for combating biofilm-forming pathogenic bacteria such as *Acanthamoeba.*	[1]
	Cellulase with pectinase	They can be used to release antioxidants from vegetable and fruit pomace, which further contribute to controlling atherosclerosis and coronary heart disease.	[2]
	Cellulase	Cellulase has been recognized as an effective agent to treat various diseases like blinding keratitis, phytobezoars, etc., by disrupting the cell wall of biofilms and plants.	[3]
	Digestin has a cellulase enzyme	Because of its high efficiency, digestin is frequently used for the digestion of cellulose fibers.	[4]

	Cellulase with chitinase and lysozyme	The synergistic action of these enzymes is used in chitosan degradation, while chitosan has multiple applications, such as bone rebuilding, anticancer agents, and encapsulation of various materials.	[5]
Bio-remediation	Immobilized cellulase	Immobilized cellulase has emerged as a biosensor in the bioremediation industry.	[6]
	Cellulase	Cellulase is regarded as a complex group of enzymes and has been used for bioremediation.	[7]
Fruit and vegetable processing	Macerating enzyme	In liquefaction, the macerating enzymes are used, which not only improve the sensory quality but also the physico-chemical characteristics of the end product.	[7]
	Cellulase, Hemi-cellulase, Pectinase	Clarification of fruit and vegetable juice was improved significantly both by cellulase alone (produced by *Paenibacillus,* and *Bacillus*) and also with a combination of cellulase with other enzymes.	[8]
	Macerating enzyme	To achieve optimal olive oil extraction with slower induction of rancidity, macerating enzymes are exploited.	[8]
	Cellulase	Cellulase-ultrasonic-assisted technology is capable of extracting flavonoids from the medicinal plant *Illicium verum*.	[9]
	Cellulase	Enzyme-assisted extraction has numerous benefits, including a higher extraction yield of the modified pigments from *Ginkgo biloba*, and is also involved in the modification of flavonoids by trans-glycosylation.	[10]
	Cellulase, pectinase	Degrade the cell walls of carrots, orange peel, and sweet potatoes, and also release the carotenoids.	[7]

	Pectinases, β-glucosidases	Infusion of these enzymes usually improves the aroma, taste sensations, and volatile characteristics of various vegetables and fruits.	[11]
Bakery process	Cellulase with hydrolytic enzymes	These enzymes lead to an improvement in bread quality and increased loaf volume.	[12]
	Cellulase, amylase, and lipase	These enzymes result in dough conditioning, which improves the flavor, increases volume after baking, and enhances shelf life.	[13]
Livestock process	Cellulase, Xylanase	Its inclusion in the hay diet improved the live weight gain of cattle.	[14]
	Fibrolytic enzymes	Feeding of fibrolytic enzymes is predicted as a promising strategy based on its ability to increase milk yield (5–25%) in cows.	[15]
	Cellulase	Improves the feed utilization by cattle, body weight gain, and milk yield.	[11]
	Trichoderma cellulase	This enzyme is used as a functional additive to improve the feed conversion ratio and the digestibility of cereal-based products.	[16]
	Bacillus cellulase	This enzyme is applied to soya grain hull degradation to enhance the nutritional value of feed for monogastric animals.	[17]
Beverage process	Amylase, Carboxypeptidase, Cellulase	Malting depends on seed germination, eventually activating cell wall degrading enzymes to make high-quality malt.	[18]
	Glucanase	This enzyme reduces the viscosity of wort and also facilitates the enhanced hydrolysis of glucan	[19]
	Cellulase	Improves the beer quality and its overall production efficiency.	[20]

	Cellulase	Enzymatic hydrolysis remarkably improves the color extraction of grapes, skin maceration, and wine quality, clarification, and stability.	[21]
	β-Glucosidases	Notably, this enzyme improves the aroma of white wine by modifying the glycosylated precursors.	[6]
Non-food	Cellulase	Cellulase improves the cleaning efficiency of detergents by reducing the redeposition of particulate soils.	[22]
	Cellulase	Acidic endoglucanase II (from *T. reesei*) and neutral cellulase enzyme (from *Humicola insolens*) have been effective candidates for biostoning.	[23]
	Cellulase	Enzymatic saccharification of lignocellulosic materials like agricultural and forest residues is carried out by cellulases for biofuel production.	[24]
	Bacterial cellulase	These enzymes from marine bivalves are applied as a pretreatment to obtain biogas from microalgae	[25]
	Cellulase	Cellulase treatment reduces fiber coarseness and decreases defibrillation.	[26]
	Cellulase	It lowers the energy needs in pulping and stickies control and increases the paper mill quality.	[27]
Agriculture area	*β*-1,3-glucanase + N-acetyl-glucosaminidase	The synergistic action of these enzymes inhibits the spore germination and also the germ tube elongation of *Botrytis cinerea.*	[28]
	Cellulase	Cellulase supplementation enhances cellulose degradation in soil. Thus, soil fertility is increased	[29]
	Paenibacillus cellulase	This enzyme might be associated with the decomposition of rhizospheric soil, which results in a significant increase in the availability of nutrients for the plant.	[30]

Antifungal agent with cellulase	Cellulase hydrolysis and antifungal compounds from *Paenibacillus ehimensis* could protect crops against pathogenic oomycetes.	[31]
Burkholderia cellulase	In particular, this enzyme can increase the internal colonization of *Vitis vinifera* roots.	[32]
Rhizobium leguminosarum cellulase	It improves the root colonization in cereal crops, thus promoting their nutritional contents and yields.	[33]

Table S2- Application of microbial enzymes in waste management

Bioremediation technique	Microbial strain	Applications	Reference
Ex-situ			
Composting	*Chaetomium thermophilum*	It plays a significant role in forming polyaromatic humic substances with phenoloxidase and peroxidase, further improving soil structure and fertility.	[33]
Composting	*Bacillus licheniformis, Trichoderma viride*	They accelerate the composting process to form safe and stable compost products enriched with humic matter.	[34]
Composting	Cellulolytic bacteria	During composting, the inoculation of cellulolytic bacteria is highly efficient and likely to improve the growth of rice (increase in rice shoot dry weight and tiller number).	[35]
Composting	*Streptomyces* spp. and arbuscular mycorrhizal fungi	Soil amendment with compost inoculated with arbuscular mycorrhizal (AM) fungi or *Streptomyces* spp. suppresses the development of bacteria in the rhizosphere of tomato plants.	[36]

Composting	*Bacillus* isolates	The composting of municipal solid waste by *Bacillus* isolates leads to a significant reduction in composting time and production of high-quality compost.	[37]
Composting	*Trichoderma harzianum, Clostridium butyricum*	Co-inoculation strategy is used to decompose wheat straw in which *T. harzianum* oxidizes carbon to CO_2 and then supplies sugar to *C. butyricum*, increasing the compost's N content.	[38]
Anaerobic digestion	*Caldicellulosiruptor bescii*	It enhances the methane production from steam-exploded birch in an anaerobic digestion, which is carried out at 62 °C.	[39]
Anaerobic digestion	*Pseudobutyrivibrio xylanivorans*	Bioaugmentation with *P. xylanivorans* is executed to escalate the methane production by 18%.	[40]
Anaerobic digestion	Lignocellulolytic microbial consortia	It enhances the lignocellulosic biomass digestibility in the anaerobic digestion of fiber-rich cellulosic substrates, including paper wastes, cassava residues, etc.	[41]
Anaerobic digestion	Mixed strain	Bioaugmentation with mixed strain increases the biogas production by 15 % during the anaerobic digestion of sweet corn processing residues than that added with a single strain.	[42]
In-situ			
Phytoremediation	*Pseudomonas sp.*	The inoculation of *Pseudomonas sp.* significantly promoted plant growth and enhanced the accumulation of nickel in the plant.	[43]
Phytoremediation	Sulfate-reducing bacteria	In FTWs, sulfur-oxidizing bacteria play a significant role in keeping the plant safe by detoxifying hydrogen sulfide.	[44]
Phytoremediation	Nitrifiers	Nitrifiers have been augmented in the aquatic root system of FTWs and are involved in the oxidation of ammonia.	[45]

Phytoremediation	*Gaeumannomyces cylindrosporus*	Inoculation with maize significantly enhanced the productivity and yield of maize under lead stress.	[46]
Phytoremediation	*Pseudomonas aeruginosa*	Its inoculation improves the plant's tolerance to cadmium stress and increases the accumulation and translocation of cadmium in the plant.	[47]
Phytoremediation	*Leptochloa fusca* with consortium	The inoculation of *Leptochloa fusca* with a consortium of three distinct endophyte bacteria in constructed wetlands raised the plant efficiency in remediating tannery effluents.	[48]
Phytoremediation	*Ralstonia eutropha*	It can reduce the harmful Cd (II) by producing metallothionein on the cell surface.	[49]
Phytoremediation	*E. coli*	It regulates the accumulated Cd toxicity by producing distinct peptides and proteins.	[50]

Reference

[1] Martinez, A.J.; Visvesvara, G.S. Free-living, amphizoic and opportunistic amebas. *Brain Pathol.* **1997**, *7*, 583–598, doi.org/10.1111/j.1750-3639.1997.tb01076.x.

[2] Meyer, A.S.; Jepsen, S.M.; Sørensen, N.S. Enzymatic release of antioxidants for human low-density lipoprotein from grape pomace. *J. Agric. Food Chem.* **1998**, *46*, 2439–2446. https://doi.org/10.1021/jf971012f.

[3] Singh, R.; Kumar, M.; Mittal, A. Microbial enzymes: industrial progress in 21st century. *3 Biotech* **2016**, *6*, 174, https://doi.org/10.1007/s13205-016-0485-8.

[4] Kuhad, R.C.; Singh, A. Biotechnology for environmental management and resource recovery, **2013**. https://doi.org/10.1007/978-81-322-0876-1.

[5] Garcia-Ubasart, J.; Torres, A.L.; Vila, C. Biomodification of cellulose flax fibers by a new cellulase. *Ind. Crops Prod.* **2013**, *44*, 71-76, doi.org/10.1016/j.indcrop.2012.10.019.

[6] Jayasekara, S.; Ratnayake, R. Microbial cellulases: an overview and applications, in: M.E.E. Martín (Ed.), IntechOpen, Rijeka, **2019**: p. Ch. 5. doi.org/10.5772/intechopen.84531.

[7] Kuhad, R.C.; Gupta, R.; Singh, A. Microbial cellulases and their industrial applications. *Enzyme Res*. **2011**, *2011*, 1–10, doi.org/10.4061/2011/280696.

[8] Dourado, F.; Bastos, M.; Mota, F.M. Studies on the properties of Celluclast/Eudragit L-100 conjugate. *J. Biotechnol.* **2002**, *99*, 121-131, doi.org/10.1016/s0168-1656(02)00178-5.

[9] Huang, D.; Zhou, X.; Si, J. Studies on cellulase-ultrasonic assisted extraction technology for flavonoids from *Illicium verum* residues. *Chem. Cent. J.* **2016**, *10*, 56, doi.org/10.1186/s13065-016-0202-z.

[10] Chen, S.; Xing, X.-H.; Huang, J.-J. Enzyme-assisted extraction of flavonoids from *Ginkgo biloba* leaves: improvement effect of flavonol transglycosylation catalyzed by *Penicillium decumbens* cellulase. *Enzyme Microb. Technol.* **2011**, *48*, 100-105, doi.org/10.1016/j.enzmictec.2010.09.017.

[11] Bhat, M.K. Cellulases and related enzymes in biotechnology. *Biotechnol. Adv.* **2000**, *18*, 355–383, doi.org/10.1016/S0734-9750(00)00041-0.

[12] Harada, O.; Lysenko, E.D.; Preston, K.R. Effects of commercial hydrolytic enzyme additives on canadian short process bread properties and processing characteristics. *Cereal Chem.* **2000**, *77*, 70–76, doi.org/10.1094/CCHEM.2000.77.1.70.

[13] Illingworth, C.D.; Cook, S.D. Acanthamoeba keratitis. *Surv. Ophthalmol.* **1998**, *42*, 493–508, doi.org/10.1016/s0039-6257(98)00004-6.

[14] Beauchemin, K.; Rode, L.; Sewalt, V. Fibrolytic enzymes increase fiber digestibility and growth rate of steers fed dry forages. *Can. J. Anim. Sci.* **1995**, *75*, 641–644. doi.org/10.4141/cjas95-096.

[15] Lewis, G.E.; Hunt, C.W.; Sanchez, W.K. Effect of direct-fed fibrolytic enzymes on the digestive characteristics of a forage-based diet fed to beef steers. *J. Anim. Sci.* **1996**, *74*, 3020–3028, doi.org/10.2527/1996.74123020x.

[16] Baker, R.A.; Wicker, L. Current and potential applications of enzyme infusion in the food industry. *Trends Food Sci. Technol.* **1996**, *7*, 279–284, doi.org/10.1016/0924-2244(96)10030-3.

[17] Wongputtisin, P.; Khanongnuch, C.; Kongbuntad, W.; Niamsup, P. Use of *Bacillus subtilis* isolates from Tua-nao towards nutritional improvement of soya bean hull for monogastric feed application. *Lett. Appl. Microbiol.* **2014**, *59*, 328–333. doi.org/10.1111/lam.12279.

[18] Gupta, R.; Mehta, G.; Sharma, S.; Jain, K.K. Cellulases and their biotechnological applications bt - biotechnology for environmental management and resource recovery, in: R.C. Kuhad, A. Singh (Eds.), Springer India, India, **2013**, 89–106. doi.org/10.1007/978-81-322-0876-1_6.

[19] Bamforth, C.W. Current perspectives on the role of enzymes in brewing. *J. Cereal Sci.* **2009**, *50*, 353–357. doi.org/10.1016/j.jcs.2009.03.001.

[20] Harman, G.E.; Kubicek, C.P. *Trichoderma* And *Gliocladium*, Volume 2: Enzymes, biological control and commercial applications, CRC Press, **1998**. https://books.google.com.pk/books?id=20NZDwAAQBAJ.

[21] Mojsov, K.; Andronikov, D.; Janevski, A.; Jordeva, S. Enzymes and wine: The enhanced quality and yield. *Savrem. Tehnol.* **2015**, *4*, 94–100. doi.org/10.5937/savteh1501094M.

[22] Maryan, A.S.; Montazer, M. A cleaner production of denim garment using one step treatment with amylase/cellulase/laccase. *J. Clean. Prod.* **2013**, *57*, 320–326. doi.org/10.1016/j.jclepro.2013.05.041.

[23] Uhlig, H. Industrial enzymes and their applications, Wiley, **1998**. https://books.google.com.pk/books?id=yhEmezjYtiUC.

[24] Nguyen, D.Q.; Mai, T.P.; Tran, T.T.A. Bioethanol production from lignocellulosic biomass, in: Y. Yun (Ed.), IntechOpen, Rijeka, **2019**, doi.org/10.5772/intechopen.86437.

[25] Muñoz, C.; Hidalgo, C.; Zapata, M.; Jeison, D. Use of cellulolytic marine bacteria for enzymatic pretreatment in microalgal biogas production. *Appl. Environ. Microbiol.* **2014**, *80*, 4199-4206. doi.org/10.1128/AEM.00827-14.

[26] Lee, S.; Park, H.; Wanhee, I. Effects of enzyme mixture and beating treatment on the properties of pulp fibers. *J. Korea Tech. Assoc. Pulp Pap. Ind.* **2020**, *52*, 101–109. https://doi.org/10.7584/JKTAPPI.2020.10.52.5.101.

[27] Yoon, L.W.; Ang, T.N.; Ngoh, G.C. Fungal solid-state fermentation and various methods of enhancement in cellulase production. *Biomass Bioenergy* **2014**, *67*, 319–338, doi.org/10.1016/j.biombioe.2014.05.013.

[28] Ajuna, H.B.; Lim, H.-I.; Moon, J.-H. The Prospect of Hydrolytic Enzymes from *Bacillus* Species in the Biological Control of Pests and Diseases in Forest and Fruit Tree Production. *Int. J. Mol. Sci.* **2023**, *24*. doi.org/10.3390/ijms242316889.

[29] Fontaine, S.; Abbadie, L. Mechanisms of the priming effect in a savannah soil amended with cellulose. *Soil Sci. Soc. Am. J.* **2004**, *68*. doi.org/10.2136/sssaj2004.0125.

[30] Han, W.; He, M. The application of exogenous cellulase to improve soil fertility and plant growth due to acceleration of straw decomposition. *Bioresour. Technol.* **2010**, *101*, 3724–3731. doi.org/10.1016/j.biortech.2009.12.104.

[31] Naing, T.; Anees, M.; Lee, Y.S.; Biocontrol of Late blight disease (hytophthora capsici) of pepper and the plant growth promotion by *aenibacillus ehimensis* KWN38. *J. Phytopathol.* **2014**, *162*, 367–376, doi.org/10.1111/jph.12198.

[32] Compant, S.; Reiter, B.; Sessitsch, A., Nowak, J. Endophytic colonization of Vitis vinifera L. by plant growth-promoting bacterium *Burkholderia* sp. strain PsJN. *Appl. Environ. Microbiol.* **2005**, *71*, 1685–1693, doi.org/10.1128/AEM.71.4.1685-1693.2005.

[33] Robledo, M.; Jiménez-Zurdo, J.I.; Soto, M.J.; Velázquez, E. Development of functional symbiotic white clover root hairs and nodules requires tightly

regulated production of rhizobial cellulase CelC2. *Mol. Plant. Microbe. Interact.* **2011**, *24*, 798–807, doi.org/10.1094/MPMI-10-10-0249.

[34] Gautam, E.; Bundela, P.S.; Pandey, A.K. Diversity of cellulolytic microbes and the biodegradation of municipal solid waste by a potential strain. *Int. J. Microbiol.* **2012,** *2012*, 325907, doi.org/10.1155/2012/325907.

[35] Lestari, Y.; Maftu'ah, E.; Annisa, W. Rice straw composting by cellulolytic bacteria isolate and its application on rice in acid sulfate soils. *BIO Web Conf.* **2020**, *20*, 1006, doi.org/10.1051/bioconf/20202001006.

[36] Badr EL-Din, S.M.S.; Attia, M.; Abo-Sedera, S.A. Field assessment of composts produced by highly effective cellulolytic microorganisms. *Biol. Fertil. Soils* **2000**, *32*, 35–40, doi.org/10.1007/s003740000210.

[37] Rastogi, M. Nandal, M. Khosla, B. Microbes as vital additives for solid waste composting. *Heliyon* **2020**, *6*, e03343, doi.org/10.1016/j.heliyon.2020.e03343.

[38] Hart, T.D.; Kinsey, G.; Kelley, J. Strategies for the isolation of cellulolytic fungi for composting of wheat straw. *World J. Microbiol. Biotechnol.* **2002**, *18*, 471–480. https://doi.org/10.1023/A:1015519005814.

[39] Mulat, D.G.; Huerta, S.G.; Kalyani, D. Enhancing methane production from lignocellulosic biomass by combined steam-explosion pretreatment and bioaugmentation with cellulolytic bacterium *Caldicellulosiruptor bescii. Biotechnol. Biofuels* **2018**, *11*, 19, doi.org/10.1186/s13068-018-1025-z.

[40] Čater, M.; Fanedl, L.; Malovrh, S. Biogas production from brewery spent grain enhanced by bioaugmentation with hydrolytic anaerobic bacteria. *Bioresour. Technol.* **2015**, *186*, 261–269. doi.org/10.1016/j.biortech.2015.03.029.

[41] Yuan, X.; Cao, Y.; Li, J.; Wen, B. Effect of pretreatment by a microbial consortium on methane production of waste paper and cardboard. *Bioresour. Technol.* **2012**, *118*, 281-288. doi.org/10.1016/j.biortech.2012.05.058.

[42] Martin-Ryals, A.; Schideman, L.; Li, P.; Wagner, N. Improving anaerobic digestion of a cellulosic waste via routine bioaugmentation with cellulolytic microorganisms. *Bioresour. Technol.* **2015**, *189*, 62-70, doi.org/10.1016/j.biortech.2015.03.069.

[43] Ma, Y.; Rajkumar, M.; Luo, Y. Inoculation of endophytic bacteria on host and non-host plants—Effects on plant growth and Ni uptake. *J. Hazard. Mater.* **2011**, *195*, 230–237. doi.org/10.1016/j.jhazmat.2011.08.034.

[44] Lamers, L.P.M.; Govers, L.L.; Janssen, I.C.J.M.; Van der Heide, T. Sulfide as a soil phytotoxin a review. *Front. Plant Sci.* **2013**, *4*, 268, doi.org/10.3389/fpls.2013.00268.

[45] Shahid, M.J.; Kouadri, F.; Ali, S.; Nawaz, N. Role of microorganisms in the remediation of wastewater in floating treatment wetlands: a review. *Sustainability* **2020**, *12*, 5559, doi.org/10.3390/su12145559.

[46] Ban, Y.; Xu, Z.; Yang, Y. Effect of dark septate endophytic fungus *Gaeumannomyces cylindrosporus* on plant growth, photosynthesis and pb tolerance of maize (Zea mays L.). *Pedosphere* **2017**, *27*, 283–292. doi.org/10.1016/S1002-0160(17)60316-3.

[47] Shi, P.; Zhu, K.; Zhang, Y. Growth and cadmium accumulation of solanum nigrum l. seedling were enhanced by heavy metal-tolerant strains of *pseudomonas aeruginosa*. *Water Air Soil Pollut.* **2016**, *227*, 459, doi.org/10.1007/s11270-016-3167-6.

[48] Ashraf, S.; Afzal, M.; Naveed, M.; Ahmad Zahir, Z. Endophytic bacteria enhance remediation of tannery effluent in constructed wetlands vegetated with

Leptochloa fusca. *Int. J. Phytoremediation* **2018**, *20*, 121–128. doi.org/10.1080/15226514.2017.1337072.

[49] Valls, M.; Atrian, S.; de Lorenzo, V.; Fernández, L.A. Engineering a mouse metallothionein on the cell surface of *Ralstonia eutropha* CH34 for immobilization of heavy metals in soil. *Nat. Biotechnol.* **2000,** *18*, 661-665, doi.org/10.1038/76516.

[50] Mejáre, M.; Bülow, L. Metal-binding proteins and peptides in bioremediation and phytoremediation of heavy metals. *Trends Biotechnol.* **2001**, *19*, 67-73. doi.org/10.1016/S0167-7799(00)01534-1.
